# Mammalian acetate-dependent acetyl CoA synthetase 2 contains multiple protein destabilization and masking elements

**DOI:** 10.1016/j.jbc.2021.101037

**Published:** 2021-07-31

**Authors:** Jason S. Nagati, Philippe H. Kobeissy, Minh Q. Nguyen, Min Xu, Trent Garcia, Sarah A. Comerford, Robert E. Hammer, Joseph A. Garcia

**Affiliations:** 1 Department of Medicine, Columbia University Medical Center, New York, New York, USA; 2 Department of Medicine, University of Texas Southwestern Medical Center, Dallas, Texas, USA; 3 Department of Molecular Genetics, University of Texas Southwestern Medical Center, Dallas, Texas, USA; 4 Department of Biochemistry, University of Texas Southwestern Medical Center, Dallas, Texas, USA; 5 Department of Research, James J. Peters VA Medical Center, New York, New York, USA

**Keywords:** acetate, acetyl-CoA synthetase, Acss2, hypoxia-inducible factor (HIF), HIF-2, protein degradation, protein stability, structure–function, transgenic mice, ABC, Acss2 basic conserved, ACS, acetyl CoA synthetase, Acss2, acetate-dependent acetyl CoA synthetase 2, CBP, Creb-binding protein, DHR, degron homology region, HIF-2, hypoxia-inducible factor 2, NLS, nuclear localization signal, MEF, mouse embryonic fibroblast, PDE, protein destabilization element

## Abstract

Besides contributing to anabolism, cellular metabolites serve as substrates or cofactors for enzymes and may also have signaling functions. Given these roles, multiple control mechanisms likely ensure fidelity of metabolite-generating enzymes. Acetate-dependent acetyl CoA synthetases (ACS) are *de novo* sources of acetyl CoA, a building block for fatty acids and a substrate for acetyltransferases. Eukaryotic acetate-dependent acetyl CoA synthetase 2 (Acss2) is predominantly cytosolic, but is also found in the nucleus following oxygen or glucose deprivation, or upon acetate exposure. Acss2-generated acetyl CoA is used in acetylation of Hypoxia-Inducible Factor 2 (HIF-2), a stress-responsive transcription factor. Mutation of a putative nuclear localization signal in endogenous Acss2 abrogates HIF-2 acetylation and signaling, but surprisingly also results in reduced Acss2 protein levels due to unmasking of two protein destabilization elements (PDE) in the Acss2 hinge region. In the current study, we identify up to four additional PDE in the Acss2 hinge region and determine that a previously identified PDE, the ABC domain, consists of two functional PDE. We show that the ABC domain and other PDE are likely masked by intramolecular interactions with other domains in the Acss2 hinge region. We also characterize mice with a prematurely truncated Acss2 that exposes a putative ABC domain PDE, which exhibits reduced Acss2 protein stability and impaired HIF-2 signaling. Finally, using primary mouse embryonic fibroblasts, we demonstrate that the reduced stability of select Acss2 mutant proteins is due to a shortened half-life, which is a result of enhanced degradation *via* a nonproteasome, nonautophagy pathway.

Acetyl CoA is a major building block of intermediary metabolism and also functions as a substrate or cofactor for enzymes including acetyltransferases, which utilize acetyl CoA as a source of acetyl groups. When acetyl CoA availability is rate-limiting, acetyl CoA generators may have signal transducing capability (1). Acetyl CoA synthetases comprise an evolutionarily conserved family of acetyl CoA generators and include acetyl CoA synthetases (ACS) or acetate-CoA ligase in prokaryotes as well as acetate-dependent acetyl CoA synthetases (Acss) in eukaryotes. Although Acss2 is considered the cytosolic member of the Acss family, it is also enriched in or translocates into the nucleus following oxygen or glucose deprivation where it has a stress signaling role through selective provision of acetyl CoA to an acetyltransferase with coactivator function (2).

Targets for Acss2-dependent acetylation include histones as well as nonhistone proteins such as Hypoxia-Inducible Factor 2 (HIF-2), a heterodimeric DNA-binding transcription factor activated by oxygen or glucose deprivation. Although subject to oxygen-dependent protein degradation, coupled acetylation and deacetylation are required for maximal HIF-2 signaling (3, 4). HIF-2 signaling is characterized by restricted interactions with other cellular factors. For example, HIF-2 acetylation is conferred by a specific acetyltransferase, Creb-binding protein (CBP), rather than its closely related homologue p300 (3, 4). Likewise, deacetylation of acetylated HIF-2 is conferred by Sirtuin 1 (3, 4) and possibly Sirtuin 2 (5). Similarly, the *de novo* source of the acetyl donor, acetyl CoA, used by CBP for HIF-2 acetylation also exhibits specificity and is provided by Acss2 rather than ATP citrate lyase (ACLY), the other *de novo* cytosolic acetyl CoA generator that also is found in the nucleus (6).

We had previously sought to generate a cytosol-restricted, enzymatically active form of Acss2. A directed mutation of a putative nuclear localization signal (NLS) in Acss2 residing in the carboxy terminal portion that changes two basic (RK) to two acidic (ED) amino acid residues abrogates HIF-2 acetylation, blunts HIF-2 dependent signaling, and impairs flank tumor growth in cancer cells expressing mutant Acss2 (7). Introduction of the Acss2 ED mutation into the mouse germline using CRISPR/Cas9 mutagenesis results in mutant mice with impaired induction of the canonical HIF-2 target gene erythropoietin and consequently blunted recovery from acute hemolytic anemia (8), similar to results obtained with Acss2 null mice (9).

In characterizing the Acss2 ED mutant mouse strain, we noted that Acss2 ED protein levels were dramatically reduced (8). Structural studies revealed that bacterial ACS protein consists of two major globular structures, an ∼80% amino portion and the remaining ∼20% carboxy portion, which contains several features required for enzymatic function (10, 11, 12). Functional studies using cell culture models identified two protein destabilization elements (PDEs) that may be active in the Acss2 ED mutant protein. One PDE corresponds to a degron identified in a screen in yeast (13), which we refer to as a degron homology region (DHR). The second PDE is just downstream from the DHR and corresponds to the putative nuclear localization signal enriched in basic residues (7, 14, 15), which we refer to as the Acss2 basic conserved (ABC) domain (8). The DHR and ABC domains are located in the carboxy terminal globular portion of mouse Acss2, also known as the hinge region (8). In this study, we sought to identify if any additional PDE resides in the Acss2 hinge region.

## Results

To identify additional elements affecting protein stability in the Acss2 hinge region, we used two types of Acss2 constructs. We introduced deletions or point mutations in full-length Acss2 protein and expressed these constructs transiently in Acss2 null primary mouse embryonic fibroblasts (MEFs). Alternatively, we generated fusion proteins consisting of isolated segments of Acss2 fused to the carboxy terminus of the fluorescent reporter moxGFP (16) and generated stable HeLa cell lines expressing these fusion proteins. With both types of constructs, we assessed steady-state protein levels by immunoblot assays. Several relevant observations were made with these constructs.

**Figure 1:**
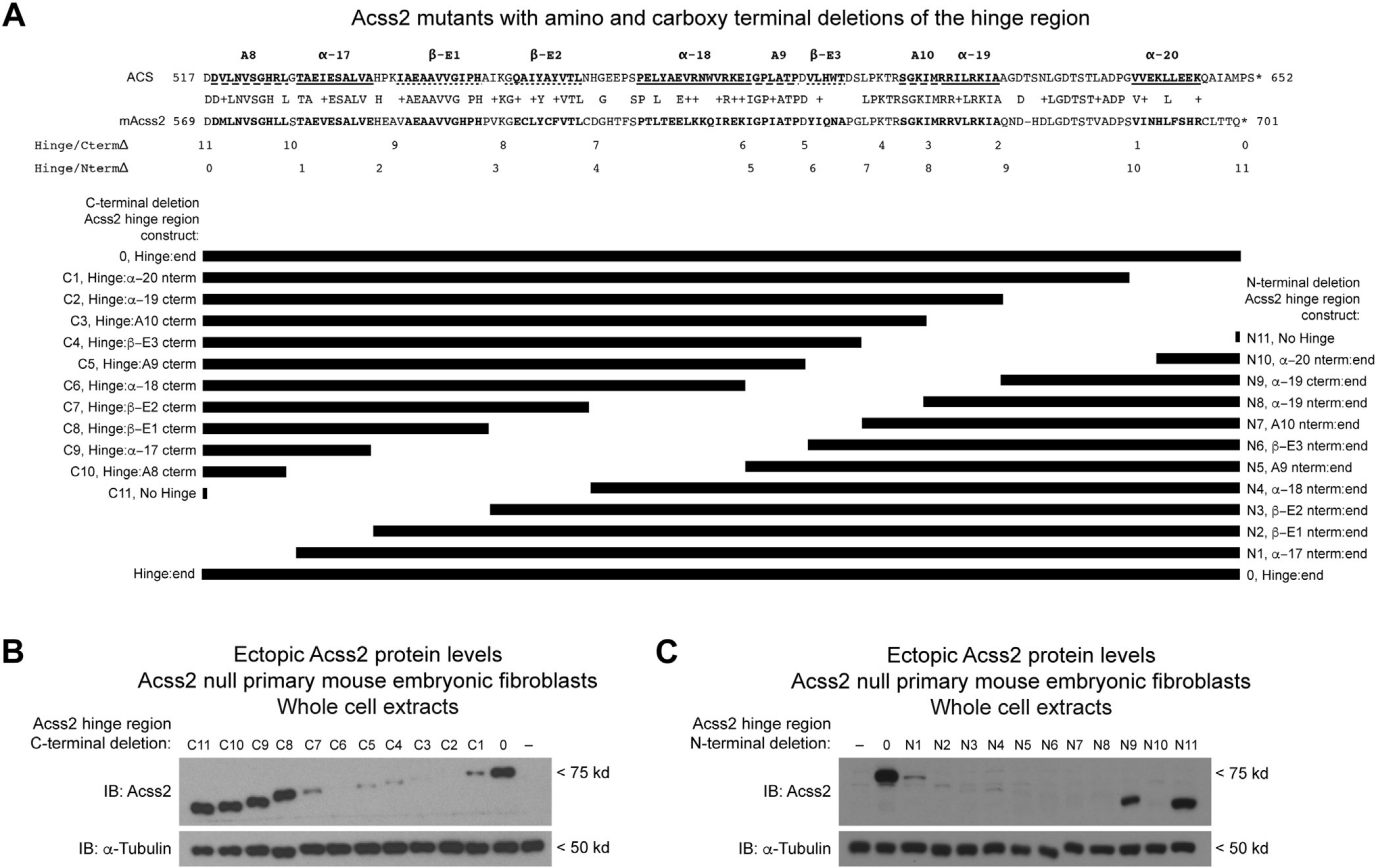
**Deletion analysis of the Acss2 hinge region using full-length Acss2 protein.***A*, alignment of prokaryotic ACS and mouse Acss2 (mAcss2) is shown with absolutely conserved residues and residues of similar properties indicated by pluses. The structural motifs designated above ACS include conserved A domains (*dashed lines*), alpha helices (*solid lines*), and beta sheets (*dotted lines*). Two series of Acss2 constructs were designed by truncations at the junctions of structural motifs from either the (*B*) carboxy or (*C*) amino terminal end of the Acss2 hinge domain and fusing the remainder to the globular portion of Acss2 upstream of the hinge domain. Each construct was introduced into Acss2 null mouse embryonic fibroblasts and protein levels were determined by immunoblot (normalized to protein amount and compared to α-tubulin).

To begin our investigations, we deleted regions from either the amino or carboxy terminus of the Acss2 hinge domain based upon structural features and fused the remaining segments to the amino terminal portion of Acss2 (Fig. 1*A*). For the Acss2 hinge domain carboxy terminal deletions (Fig. 1*B*), removal of α − 20 through α − 18 destabilized the Acss2 mutant protein (Fig. 1*B*, constructs C1-C7). Further deletion of β-E2 stabilized the remaining protein (Fig. 1*B*, construct C8), which was unaffected by removal of the remaining upstream elements of the hinge region (Fig. 1*B*, constructs C9–C11).

For the Acss2 hinge domain amino terminal deletions (Fig. 1*C*), removal of A8 through A10, which truncates the ABC domain mid-way through its sequence, resulted in reduced protein stability (Fig. 1*C*, constructs N1–N8). Deleting the remainder of the ABC domain resulted in a stable Acss2 mutant protein (Fig. 1*C*, construct N9). Interestingly, deletion of the segment between α − 19 and α − 20 yielded an unstable Acss2 mutant protein (Fig. 1*C*, construct N10). Removal of the remaining α − 20 segment stabilized the Acss2 mutant protein, which consists solely of the amino globular domain comprising the first ∼80% of the native Acss2 protein (Fig. 1*C*, construct N11).

**Figure 2:**
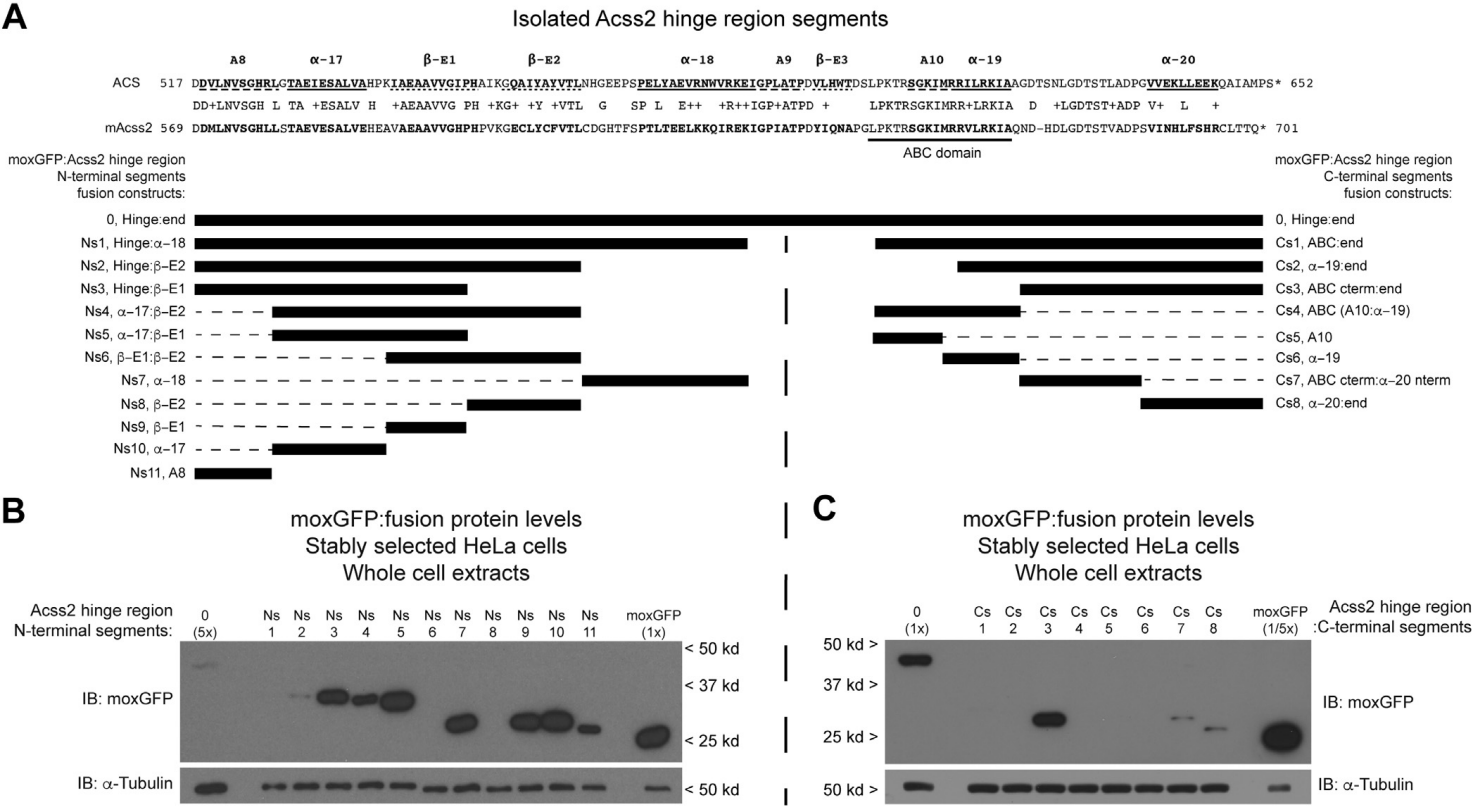
**Deletion analysis of the Acss2 hinge region using moxGFP fusion proteins**. *A*, lentivirus expressing moxGFP fused to various segments of the Acss2 hinge domain (*black boxes*) shown relative to the alignment of ACS and mAcss2 were used to stably transform HeLa cells. Whole cell extracts prepared from HeLa stable cell lines expressing moxGFP fused at its carboxy terminus with (*B*) amino or (*C*) carboxy localized regions of the Acss2 hinge region were assayed for fusion protein expression by immunoblot (normalized to protein amount and compared with α-tubulin). Because several moxGFP:Acss2 fusion proteins in the amino-localized Acss2 series have similar stability as the parental moxGFP, the moxGFP:Acss2 hinge fusion protein was loaded at 5× level of the other fusion proteins for comparison. Because the parental moxGFP fusion protein level was substantially higher than all moxGFP:Acss2 fusion proteins in the carboxy-localized Acss2 series, the parental moxGFP alone stable cell line was loaded at one-fifth that of the other fusion protein cell lines for comparison.

The results observed from these initial experiments are consistent with the DHR and ABC domain functioning as independent PDE (8). However, these results also suggested the existence of additional PDE as well as masking domains in the Acss2 hinge region. To further investigate, we assessed the stability of heterologous fusion proteins that link moxGFP to amino or carboxy localized segments of the Acss2 hinge region (Fig. 2*A*). With respect to amino localized segments, the moxGFP:A8 fusion protein exhibited modest PDE activity (Fig. 2*B*, construct Ns11). Deletion of the A8 segment increased stability consistent with A8 functioning as a PDE (Fig. 2*B*, construct Ns3 versus construct Ns5). However, the carboxy terminal deletion of full-length Acss2 retaining A8 was stable (Fig. 1*B*, construct C10), indicating that A8 does not function as a PDE in this context. Furthermore, isolated deletion of the A8 region resulted in an unstable Acss2 protein (Fig. 1*C*, construct N1), suggesting a masking function of the A8 domain. Thus, A8 exhibits both PDE and masking activity, depending upon the type of mutant protein assay employed.

With respect to other amino-localized segments, the moxGFP fusion protein with β-E2 alone was highly unstable (Fig. 2*B*, construct Ns8) as were moxGFP fusion proteins containing β-E2 compared with those without this segment (Fig. 2*B*, constructs Ns2 *versus* Ns3, Ns4 *versus* Ns5, Ns6 *versus* Ns9). In conjunction with our initial data (Fig. 1*B*, constructs C7 *versus* C8), we conclude that β-E2 is a potent PDE. Several amino-localized segments fused in isolation with moxGFP were highly stable including α − 17, β-E1, and α − 18 (Fig. 2*B*, constructs Ns10, Ns9, Ns7). The moxGFP:α − 17 fusion protein was particularly stable and the presence of α − 17 partially offsets instability conferred by β-E2 domain (Fig. 2*B*, constructs Ns4 *versus* Ns6). However, the presence of these segments is not sufficient to override multiple PDE in deletion constructs of full-length Acss2 (Fig. 1*C*, constructs N1–N4). In conjunction with earlier data, we conclude that A8 has both PDE and masking capacities, β-E2 is a novel PDE, and α − 17 may function as a stabilizing or masking domain in the hinge region.

Immunoblots of whole cell extracts prepared from stably transformed HeLa cells expressing carboxy terminal fusions revealed that this region contains multiple PDE (Fig. 2*C*). As we previously reported (8), the isolated ABC domain was a potent PDE (Fig. 2*C*, construct Cs4). Notably, the two segments resulting from partitioning of the ABC domain, A10 and α − 19, retained potent protein destabilization capacity (Fig. 2*C*, constructs Cs5 and Cs6). The region downstream of the ABC domain did not function as a PDE when fused in its entirety to moxGFP (Fig. 2*C*, construct Cs3), similar to the chimeric Acss2 protein consisting of this region fused to the amino globular portion of Acss2 (Fig. 1*C*, construct N9). However, further partitioning of this region revealed the presence of two functional PDE (Fig. 2*C*, constructs Cs7 and Cs8), which include the α − 20 segment identified earlier in fusions with the amino globular portion of Acss2 (Fig. 1*C*, construct N10). Thus, these two PDEs remain masked when present together in a contiguous manner.

**Figure 3:**
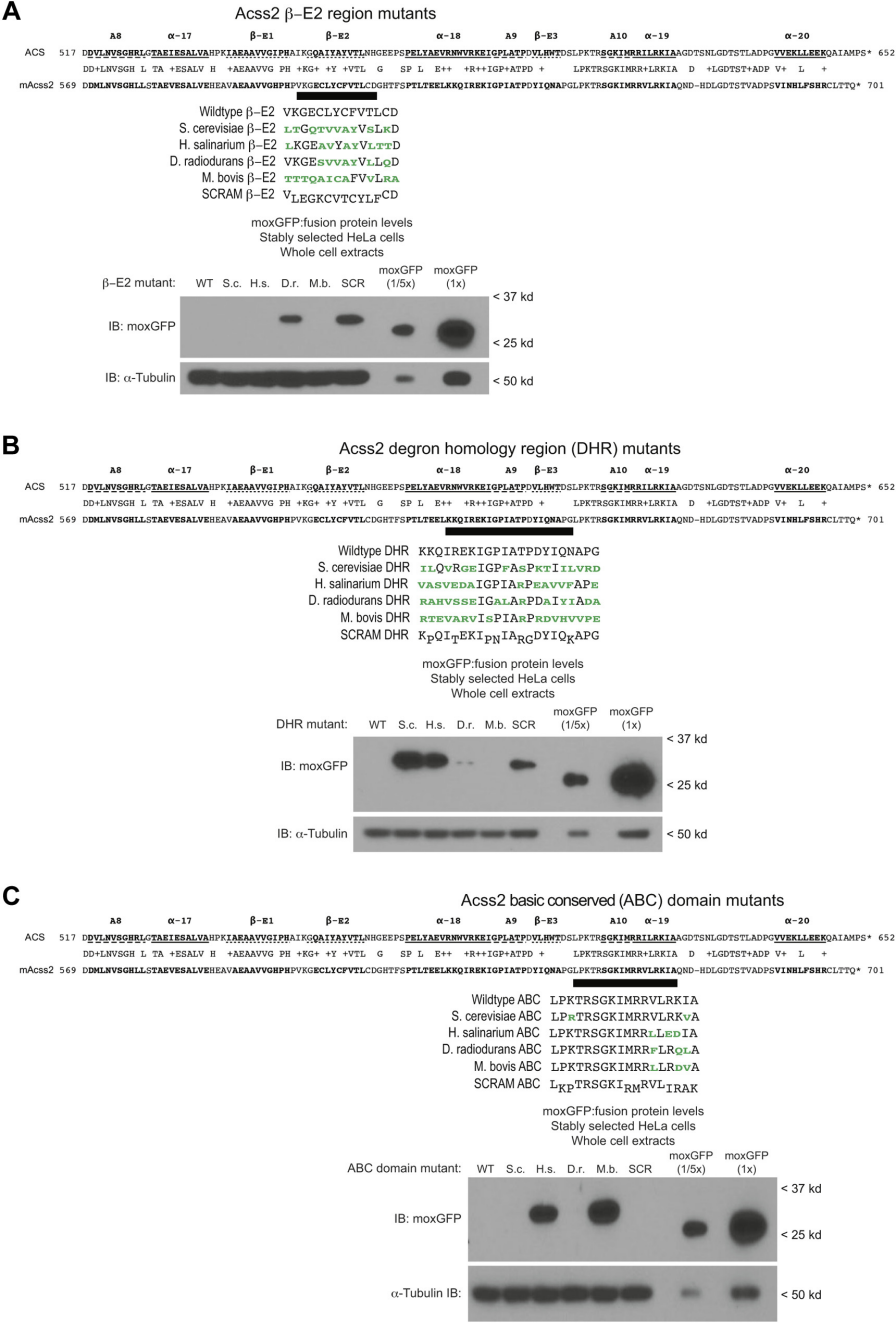
**Sequence requirements of PDE identified from cross-species substitutions**. To evaluate for sequence-specific versus structural-specific determinants that may dictate PDE ability, segments corresponding to the (*A*) E2, (*B*) DHR, and (*C*) ABC domain from mouse Acss2 (mAcss2) were identified from a lower eukaryote (*S. cerevisiae*) or several prokaryotes (*H. salininarium*, *D. radiodurans*, *M. bovis*). These elements as well as a scrambled mutant (SCRAM) that retains the amino acid composition, but disrupts the order, were fused to moxGFP and compared with the mouse Acss2 PDE or moxGFP alone as references in immunoblot analyses with whole cell extracts obtained from stably transformed HeLa cells. Amino acids from other organisms that differ from mAcss2 (*green text*) and residues at altered positions in the scrambled mutants (*down-set text*) are indicated. Because the parental moxGFP fusion protein level was substantially higher than all moxGFP:Acss2 fusion proteins in these series, the parental moxGFP alone stable cell line was also loaded at one-fifth that of the other fusion protein cell lines for comparison.

We next sought to assess if there are sequence or structural determinants that confer an ability of the β-E2 region, DHR, and ABC domain to function as PDE. To start, we identified similar regions from a lower eukaryote (*S. cerevisiae*) or from several prokaryotes (*H. salininarium, D. radiodurans, M. bovis*) and fused these to moxGFP (Fig. 3, *A*–*C*). Our assumption was that these elements from Acss2 homologues of diverse organisms are likely to have similar structural features, even though their primary amino acid sequence varies. In the event that overall charge or other physical properties confer PDE activity, we also generated a scrambled mutant (SCRAM) for each PDE that retained the amino acid composition, but disrupted the primary sequence order. These alternative elements were compared to the mouse Acss2 PDE and parental moxGFP in immunoblot analyses.

For the β-E2 constructs (Fig. 3*A*), the β-E2 element from *D. Radiodurans* does not function as an effective PDE and the SCRAM mutant likewise loses PDE function. For the DHR constructs (Fig. 3*B*), DHR elements from two organisms as well as the SCRAM mutant exhibit reduced PDE function. Interestingly, the corresponding DHR element from *S. cerevisiae* lacks PDE activity. For the ABC domain constructs (Fig. 3*C*), ABC domains from two organisms lack PDE function including that from *H. salinarium*, which contains an acidic glutamic acid–aspartic acid (ED) substitution for a basic arginine–lysine pair (R668, K669), and that from *M. bovis*, which substitutes an aspartic acid residue for a lysine (K669). However, the initial SCRAM mutant for the ABC domain remained a potent PDE, even though it altered the positions of several basic residues.

**Figure 4:**
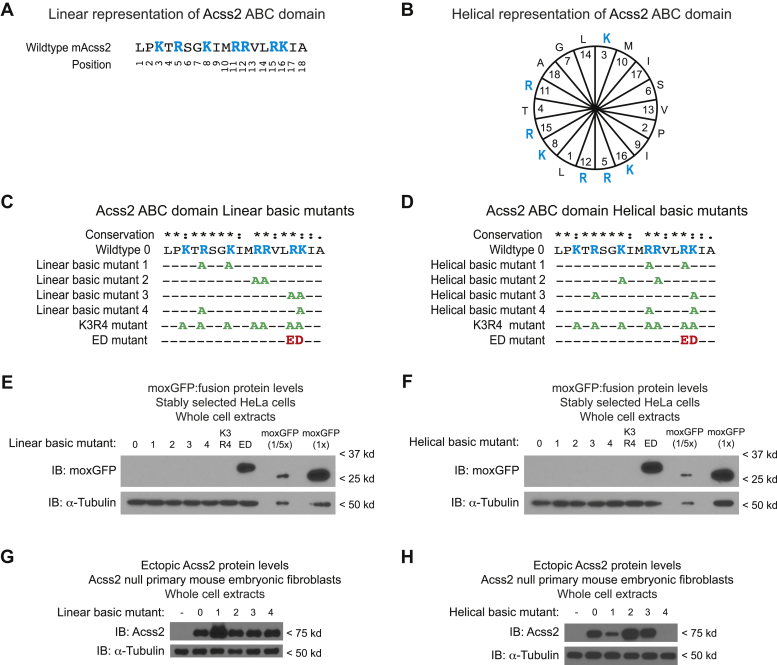
**Alanine substitutions for ABC domain basic residues do not affect its PDE function**. The ABC domain contains multiple basic amino acid residues in proximity to each other whether viewed in a (*A*) linear or (*B*) helical manner. To assess whether basic charge density dictates PDE ability for the ABC domain, fusions of moxGFP with isolated ABC domains containing pairwise alanine substitutions for basic residues adjacent to each other or at the extreme ends when viewed in either the (*C*) linear or (*D*) helical perspective were constructed. The linear basic mutant 4 is identical to helical basic mutant 3, whereas all remaining mutants are unique. Levels of moxGFP fusion proteins containing the isolated ABC domain from (*E*) linear or (*F*) helical mutants were compared with levels of a pan-basic residue alanine substitution mutant (K3R4), parental Acss2 WT ABC domain fusion, Acss2 ED mutant ABC domain fusion, or moxGFP alone in immunoblot analyses with whole cell extracts obtained from stably transformed HeLa cells. Similar (*G*) linear and (*H*) helical mutations were generated in full-length Acss2 and used to examine protein stability when expressed in Acss2 null mouse embryonic fibroblasts. The numbering in the helical representation corresponds to the positions in the linear representation. Basic residues (*blue text*), acidic ED substitution (*red text*), and alanine substitutions (*green text*) are indicated. To demonstrate the effect of the ED mutation on ABC domain PDE activity, the parental moxGFP alone stable cell line was loaded at one-fifth as well as equal levels of the other fusion protein cell lines for comparison.

Because the ABC domains from *H. salinarium* and *M. bovis* exhibited reduced PDE function, which contain at least one acidic amino acid substitution for a basic residue, we considered the possibility that basic charged residues may be important for the ABC domain to function as a PDE. The primary sequence of the ABC domain is enriched in basic residues (Fig. 4*A*). The secondary structure of the ABC domain contains an alpha-helix with basic residues biased toward one surface (Fig. 4*B*). We introduced pairwise alanine substitutions of basic residues in the ABC domain, visualized as a linear (Fig. 4*C*) or amphipathic alpha-helical (Fig. 4*D*) structure, in moxGFP:ABC domain fusion proteins (Fig. 4, *E* and *F*) as well as in full-length Acss2 protein (Fig. 4, *G* and *H*). We also examined a mutant Acss2 protein containing alanine substitutions for all basic residues (K3R4) or one containing the ED substitution mutation based upon the *H. salinarium* sequence. Isolated ABC domain:moxGFP fusion proteins containing alanine substitutions for all basic residues retained their ability to function as a PDE. In comparison, most of the full-length Acss2 alanine substitution mutants were stable. However, two helical pairwise alanine substitution mutants exhibited reduced stability compared with the parental Acss2 protein. These two mutants, [R664A, R668A] and [R664A, K669A] (Fig. 4*H*, constructs 1 and 4), have one residue in common, R664.

**Figure 5:**
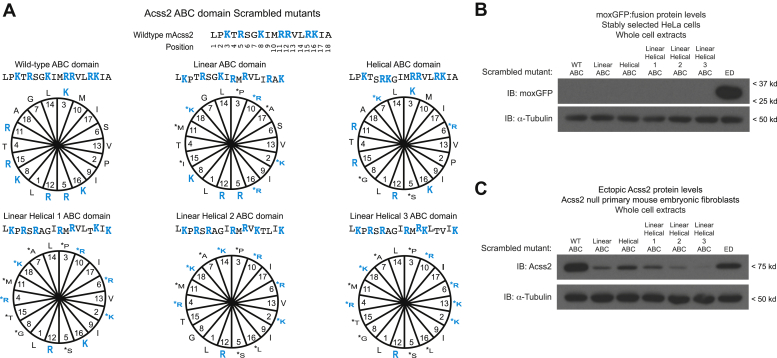
**Scrambled mutations of ABC domain basic residues do not affect its PDE function**. *A*, to further assess for a linear sequence requirement for PDE function in the ABC domain, a series of mutants were examined that varied positions of basic residues viewed in a linear or helical manner. The numbering in the helical representation corresponds to the positions in the linear representation. Basic residues (*blue text*) and residues at altered positions (*down-set text*) are indicated. *B*, these constructs were fused to moxGFP and compared with the mouse Acss2 WT and Acss2 ED mutant ABC domain fusion proteins in immunoblot analyses with whole cell extracts obtained from stably transformed HeLa cells. *C*, similar mutations were generated in full-length Acss2 and used to examine protein stability when expressed in Acss2 null mouse embryonic fibroblasts.

The results of the alanine substitutions indicated that a subset of basic residues may be important for stability of the full-length Acss2 protein. To further address determinants that confer PDE function to the ABC domain, we made five scrambled mutants of the ABC domain that varied its primary sequence (Fig. 5*A*) while retaining the overall amino acid composition and varied the charge distribution of the basic residues when viewed in a linear and/or amphipathic helix representation. Notably, all scrambled mutants remained potent PDE when examined as isolated moxGFP:ABC domain fusions (Fig. 5*B*). Furthermore, and in contrast to the alanine substitutions (Fig. 4), all scrambled mutations resulted in destabilization of the full-length Acss2 protein (Fig. 5*C*). The results from the pairwise alanine substitutions for basic residues and the scrambled mutations of the ABC domain in total indicated that specific residues in the ABC domain may be important for Acss2 protein stability.

**Figure 6:**
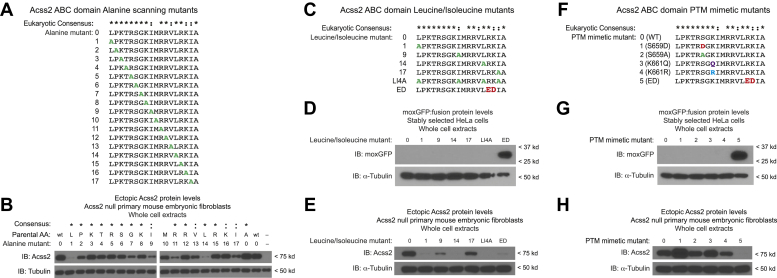
**Specific residues in the ABC domain are required for Acss2 protein stability**. *A*, to further assess for determinants of Acss2 protein stability, alanine scanning mutants (*green text*) were generated in the ABC domain of full-length Acss2. *B*, the scanning mutants were expressed in Acss2 null mouse embryonic fibroblasts (MEF) to assess protein stability. *C*, constructs were generated with alanine substitutions (*green text*) for each or all of four leucine/isoleucine residues in the ABC domain. These were compared with mouse Acss2 WT and ED (*red text*) mutant ABC domain fusion proteins. *D*, the leucine/isoleucine isolated ABC domains were fused to moxGFP and compared with mouse Acss2 WT and ED mutant ABC domain fusion proteins in immunoblot analyses with whole cell extracts obtained from stably transformed HeLa cells. *E*, similar alanine substitutions for the ABC domain leucine/isoleucine residues were generated in full-length Acss2 and compared with mouse Acss2 WT and ED mutant for protein stability in Acss2 null MEF. *F*, to assess whether posttranslational modifications (PTM) of residues in the ABC domain might affect PDE function, phosphorylation (S659D, *red text*), dephosphorylation (S659A, *green text*), acetylation (K661Q, *purple text*), and deacetylation (K661R, *blue text*), substitution mimetic mutants were generated in previously reported phosphorylation (S659) and acetylation (K661) sites. *G*, PTM mimetic constructs were fused to moxGFP and compared with mouse Acss2 WT and ED mutant ABC domain fusion proteins for protein stability in stably transformed HeLa cells. *H*, similar PTM mimetic mutations were generated in full-length Acss2 and used to examine protein stability in Acss2 null MEF.

To assess for the contribution of individual amino acid residues in the ABC domain to Acss2 protein stability, we generated additional mutants of full-length Acss2 protein using alanine scanning mutagenesis (Fig. 6*A*). Substitutions for select residues had significant effects on stability of the full-length Acss2 protein with the most drastic changes occurring with substitutions of three of four leucine and isoleucine residues (Fig. 6*B*). We examined the sequences of the scrambled ABC domain mutants and noted that they all retained at least three of these four leucine and isoleucine residues in their native positions (Fig. 5*A*). We constructed moxGFP:ABC domain fusions and full-length Acss2 expression constructs with single alanine substitutions for these residues as well as a construct with alanine substitutions for all four leucine and isoleucine residues (Fig. 6*C*). Alanine substitutions for any or all leucine or isoleucine residues in the ABC domain did not increase stability of the moxGFP:ABC domain fusion proteins (Fig. 6*D*). Similar to the scrambled ABC domain mutants, in the context of full-length Acss2 protein, these mutations resulted in instability with two of the isolated substitutions mutants and the pan-substitution mutant having a comparable or greater effect as the ED substitution (Fig. 6*E*).

We considered the possibility that posttranslational modifications (PTMs) of amino acid residues in the ABC domain might regulate Acss2 protein stability. Other investigators have shown that the serine at position 659 and the lysine at position 661 undergoes phosphorylation (15) and acetylation (17, 18), respectively. We therefore constructed a series of substitution mutants of the ABC domain that serve as PTM mimetics including S659D and S659A, which mimic the phosphorylated and dephosphorylated states, as well as K661Q and K661R, which mimic the acetylated and deacetylated states (Fig. 6*F*). The moxGFP:ABC domain fusion proteins containing these mutations had reduced stability relative to moxGFP alone or to the more stable mutant moxGFP:ED ABC domain fusion protein, similar to the moxGFP WT ABC domain protein (Fig. 6*G*). Full-length Acss2 proteins containing these mutations exhibited comparable or increased stability relative to WT Acss2 protein (Fig. 6*H*).

The results of the mutational analyses in summary indicate that three elements function as potent PDE in isolation: the β-E2 element, the DHR, and the ABC domain. Amino acid substitutions can reduce the PDE capability of each significantly. The ABC domain was unique in that the isolated ABC domain was resistant to changes in the primary or secondary sequence generated by scramble mutations as well as by alanine substitutions for all basic residues. However, there was discordance when alanine substitution mutants were expressed in the context of full-length Acss2 as only a subset of alanine substitution mutants exhibited instability. In contrast, acidic substitutions for select basic residues as present in the *M. Bovis* ABC domain, *H. salinarium* ABC domain, and the CYT (ED) substitution mutant based on the latter inactivate PDE function of the isolated ABC domain, yet full-length ED Acss2 protein were unstable. These data in total suggested that the ABC domain and other PDE might be normally masked and that masking may depend upon specific basic residues located at precise positions in the ABC domain.

**Figure 7:**
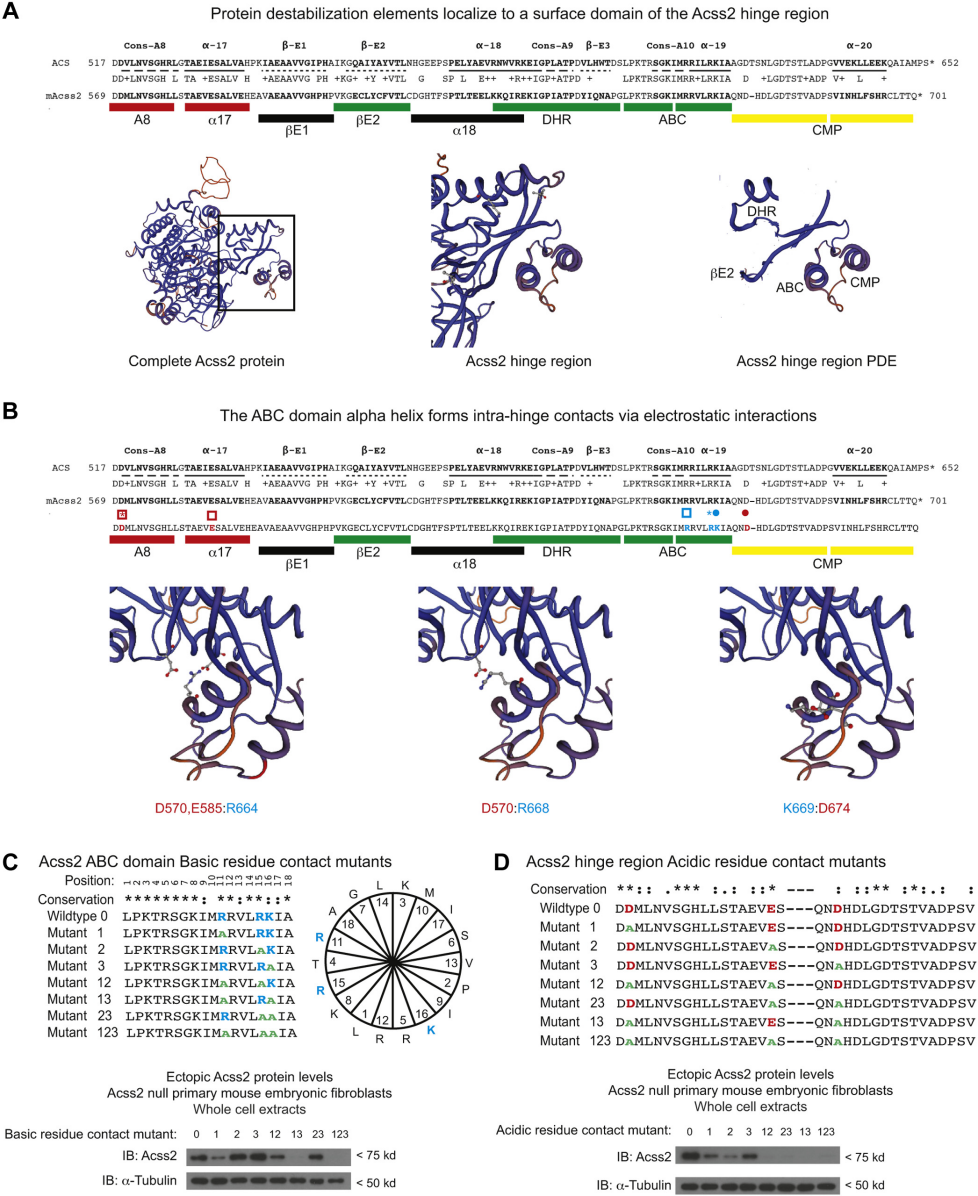
**Acss2 PDEs are in close proximity to each other in the Acss2 hinge region**. *A*, location of masking elements and PDE in the Acss2 hinge domain is shown relative to ACS and mAcss2 proteins. A8 and α17 function as masking elements (*red bars*). The PDE (*green bars*) include E2, DHR, and ABC domain with the latter consisting of two functional PDE. The conditionally masked PDE (CMP; *yellow bars*) is stable when present together, whereas individual segments confer instability. SwissProt modeling predicts that all three PDEs are in close proximity on the Acss2 carboxy hinge domain surface. *Panels* from *left* to *right* represent the entire Acss2 protein, close-up of the Acss2 hinge region containing the PDE, and close-up of the PDE alone. *B*, acidic (*red*) and basic (*green*) residues in the Acss2 hinge region that may form electrostatic interactions are shown with *boxes*, *dots*, and *asterisks* indicating matching participants for each interaction. SwissProt modeling is also shown for each predicted interaction. The effect on protein stability of full-length Acss2 protein expressed in Acss2 null mouse embryonic fibroblasts of single, double, or triple alanine substitutions for the three (*C*) basic (*blue text*) or (*D*) acidic (*red text*) residues that comprise the electrostatic interactions is shown with alanine substitutions (*green text*).

In the deletion mutational analyses, we identified at least three masking elements present in the hinge region: the A8 domain, the α − 17 domain, and the region downstream of the ABC domain. Because the hinge region forms a globular structure in crystal structures of prokaryotic ACS proteins, we reasoned that masking might be facilitated if the PDEs are in close proximity to each other. To determine if this might be the case, we used a web-based modeling prediction program to map PDE on the predicted tertiary structure of Acss2 (Fig. 7*A*). Indeed, all PDEs are predicted to be in close proximity to each other on the surface of the hinge domain and in a globular portion spatially distinct from the major body of the Acss2 protein.

We next asked if the PDE might be hidden by recruitment of masking elements we had defined in our mutational analyses. Using the web-based modeling program (Fig. 7*B*), we identified three basic residues in the ABC domain (R664, R668, K669) that form potential electrostatic interactions with three acidic residues located in A8, α − 17, and the region downstream of the ABC domain (D570, E585, D674). To evaluate whether these residues have a functional role in preventing PDE exposure and therefore Acss2 protein instability, we performed single, double, or triple alanine substitutions for the basic (Fig. 7*C*) and acidic residues (Fig. 7*D*) and assessed their effect on stability of full-length Acss2 protein. The results indicated that at least one double and all triple alanine substitutions for either basic or acidic contact points result in marked Acss2 protein instability.

We asked if the residues identified as potential contact points are evolutionarily conserved. We obtained protein sequences for Acss2 and its homologues from the NCBI Protein and UniProt Database. The majority of Acss2 protein sequences currently in the database are *in silico* predictions based upon whole genome sequencing. Because the genomic sequences used in this predictions have not been curated to the same degree as commonly used model organisms, we first performed a quality assessment protocol whenever *in silico* protein sequences deviated significantly from the consensus protein sequence for Acss2 (eukaroytes) or ACS (prokaryotes) to ascertain if sequences aligned better when potential frame-shift errors were corrected. Final edited protein sequences were aligned and compared using Clustal Omega (19, 20).

**Figure 8:**
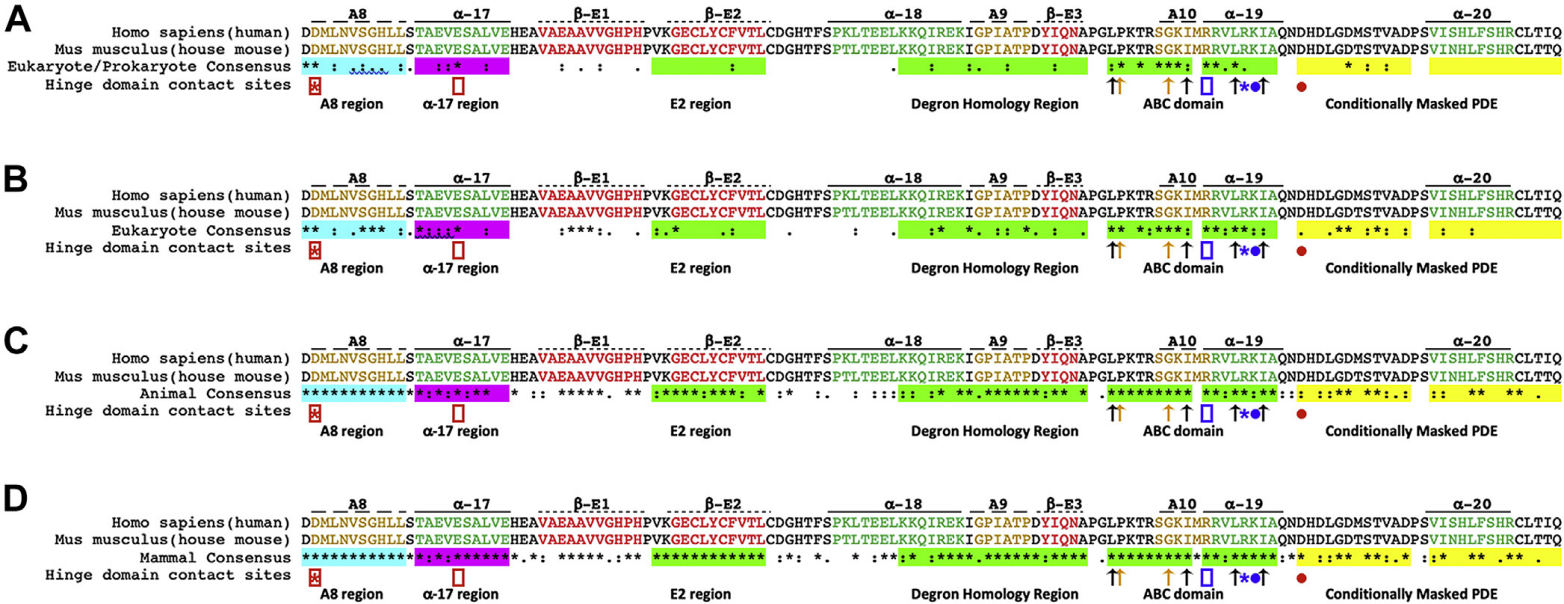
**Protein stabilizing residues are conserved in the Acss2 hinge region**. Alignment of the hinge regions of ACS and Acss2 proteins using Clustal Omega is shown with mouse Acss2 (NCBI Reference Sequence NP_062785.2) and human ACSS2 (NCBI Reference Sequence NP_001070020.2) as references for (*A*) Eukaryote and Prokaryote, (*B*) Eukaryote, (*C*) Animal, and (*D*) Mammal clusters. Structural features from prokaryotic ACS studies include conserved A elements (*gold text*, highlighted with a *dashed line*), alpha helical elements (*green text*, highlighted with a *solid line*), and beta sheet elements (*red text*, highlighted with a *dotted line*). Residues conserved across all organisms (∗), or between groups of amino acids with strongly (:) or weakly (.) similar properties are indicated. The protein destabilization elements (PDE, *shaded green*) include the E2 region, Degron Homology Region (DHR), and Acss2 basic conserved (ABC) domain. The dual PDE/masking A8 region (*shaded blue*), α17 masking domains (*shaded magenta*), and conditionally masked PDE (CMP, *shaded yellow*) are also indicated. Separate segments within the ABC domain and CMP indicate that two functional PDE were identified within each of these elements. The protein stabilizing residues include electrostatic contact sites (*boxes*, *asterisks*, and *circles* with *red* and *blue* highlighting acidic and basic residues, respectively) and the noncharged leucine/isoleucine residues (*black arrows*) as well as proline and glycine residue (*orange arrows*) residues in the ABC domain identified by alanine scanning mutagenesis.

The alignment results indicated that two basic contacts in the ABC domain and their acidic contacts in the A8 and α − 17 elements are conserved across prokaryotes and eukaryotes (Fig. 8). The third basic residue as well as its acidic contact is highly or completely conserved in eukaryotes, chordates, and mammals. Of note, this acidic residue is located in the region downstream of the ABC domain, which comprises the CMP for mouse Acss2 and is varied in length for prokaryotic ACS. Nevertheless, although less conserved, there is overall a relative enrichment in acidic residues in this region of ACS proteins. Thus, other acidic residues in this region downstream of the ABC domain may function as an acidic contact partner for a basic residue in the ABC domain of ACS proteins. Of note, the leucine, isoleucine, proline, and glycine residues in the ABC domain that are critical for stability of full-length Acss2 protein as assessed by alanine scanning mutagenesis (Fig. 6, *A* and *B*) are also highly conserved in Acss2 and its homologues.

**Figure 9:**
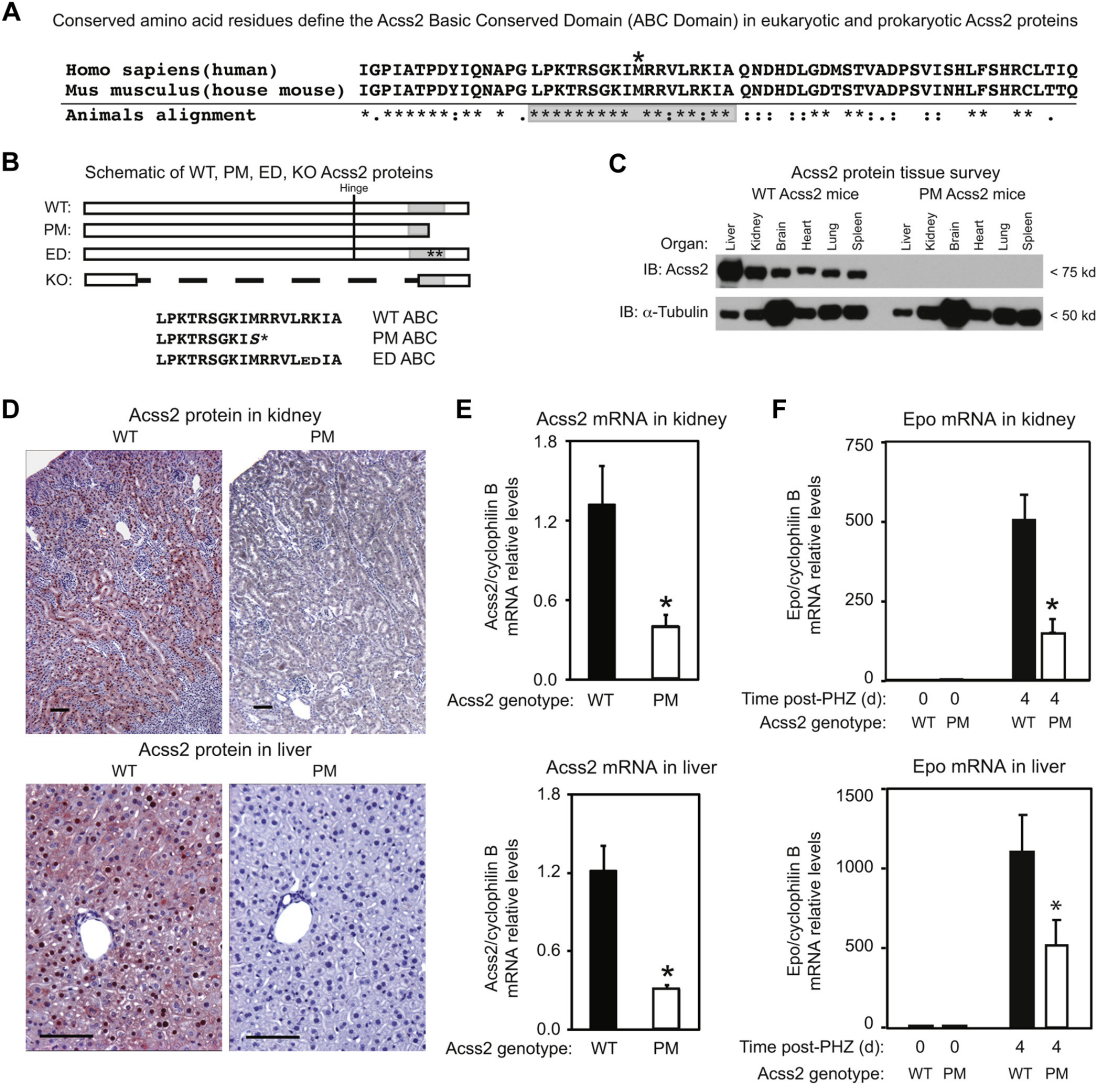
**A truncation mutation lowers Acss2 protein levels and impairs HIF-2 signaling**. *A*, alignment of the ABC domain and surrounding sequence in human and mouse Acss2 relative to conserved residues in animals. Residues conserved across all organisms (∗), or between groups of amino acids with strongly (:) or weakly (.) similar properties are indicated. The *asterisk* above human ACSS2 and mouse Acss2 denotes the location of the PM truncation mutation. *B*, schematic of Acss2 in Acss2 WT, PM, ED, and KO mice with the hinge residue indicated by a vertical line and the ABC domain by a *gray box*. The amino acid sequence for the ABC domain in Acss2 WT, PM, and ED mice is shown with the additional residue in Acss2 PM (S) or the substituted residues in Acss2 ED (ED for RK) protein indicated. *C*, mice expressing Acss2 PM have undetectable protein levels in an immunoblot tissue survey. *D*, Acss2 protein assessed by immunohistochemistry in the kidney and liver of Acss2 PM mice is minimal or undetectable, respectively. Black lines represent 100-micron scale bars. *E*, real-time PCR measurements reveal reduced Acss2 mRNA levels in Acss2 PM relative to Acss2 WT kidney (∗*p* = 0.012 for one-tail *t* test) and liver (∗*p* = 0.001 for one-tail *t* test). *F*, Real-time PCR measurements reveal blunted Epo expression in anemic Acss2 PM relative to Acss2 WT kidney (∗*p* = 0.002 for one-tail *t* test) and liver (∗*p* = 0.033 for one-tail *t* test). Data is mean with SEM with n = 8 mice per group for all groups.

We have shown that the Acss2 ED mutation has *in vivo* consequences for Acss2 protein stability and HIF-2 signaling (8). The results from our cell culture studies indicate that nonsense and other missense variants may also result in loss-of-function phenotypes by reducing Acss2 protein levels. While generating Acss2 ED mutant mice, we identified a mutant strain with a truncating point mutation (PM) in the ABC domain (Fig. 9, *A* and *B*), which results in a carboxy terminal deletion protein similar to one that was unstable in our cell culture experiments(construct N8, Fig. 1*C*). As predicted, the Acss2 protein generated in Acss2 PM mutant mice is undetectable in a tissue survey (Fig. 9*C*). When assessed by immunohistochemistry, Acss2 PM protein is present at minimal or undetectable levels in mouse kidney and liver, respectively (Fig. 9*D*). Similar to Acss2 ED mice (8), Acss2 mRNA levels are reduced in Acss2 PM kidney and liver (Fig. 9*E*).

Because of the role of Acss2 in HIF-2 signaling, we asked if Acss2 PM mutant mice had defects in HIF-2-dependent signaling. Acute hemolytic anemia following injection of phenylhydrazine (PHZ) activates HIF-2 signaling in mice. Hematocrit levels in Acss2 WT and PM mice were similar at baseline (mean with SEM; n = 8 mice per group; WT = 48.3 ± 1.2, PM = 50.0 ± 2.0). WT and PM mice treated with PHZ and with similar degrees of anemia (mean with SEM; n = 8 mice per group; WT = 21.9 ± 1.7, PM = 22.6 ± 1.9) were chosen for comparison. Gene expression for erythropoietin (Epo), a HIF-2 selective target gene, was blunted in the kidney as well as liver (Fig. 9*F*), sites of endocrine Epo synthesis in mammals, in anemic Acss2 PM mice relative to anemic Acss2 WT mice.

**Figure 10:**
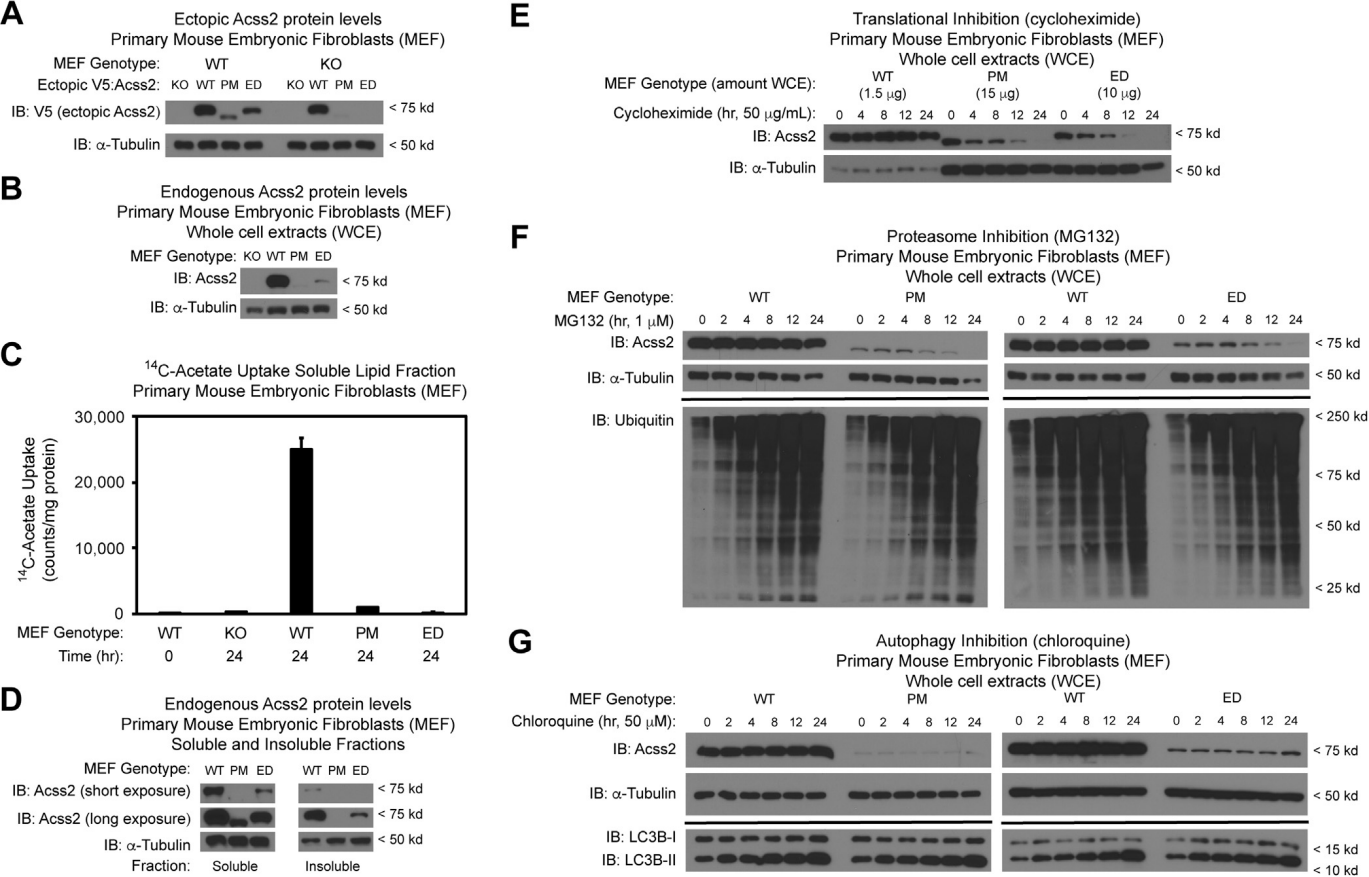
**Truncating and substitution mutations in Accs2 reduce half-life and impede function.***A*, transient transfection of Acss2 WT or KO mouse embryonic fibroblasts (MEFs) with Acss2 WT, PM, or ED expression vectors reveals lower ectopic Acss2 PM and ED protein levels relative to ectopic Acss2 WT protein. *B*, Acss2 protein levels are lower in MEF derived from Acss2 PM and ED mice compared with MEF derived from Acss2 WT mice. *C*, Acss2 KO, PM, and ED MEF have reduced acetate-derived lipid synthesis relative to WT MEF. *D*, ratios of soluble and insoluble Acss2 WT, PM, or ED protein in MEF derived from the respective mouse strain are similar. *E*, Acss2 PM and ED proteins have reduced half-lives compared with Acss2 WT protein in MEF derived from the respective mouse strain using following inhibition of translational initiation with cycloheximide. *F*, Acss2 PM and ED protein levels do not increase following inhibition of ubiquitin-mediated proteasome degradation with MG132. *G*, Acss2 PM and ED protein levels do not increase following inhibition of autophagy with chloroquine.

We asked if the effect of the Acss2 PM and ED mutations is cell-intrinsic. Transient expression of Acss2 PM protein in Acss2 WT or KO MEF resulted in ectopic Acss2 PM protein levels significantly lower than ectopic WT Acss2 protein and even lower than ectopic ED Acss2 protein expressed in a similar manner (Fig. 10*A*). Interestingly and as observed with ectopic mutant Acss2 protein expressed in transformed stable cell lines, ectopic PM and ED Acss2 protein levels were higher when expressed in WT versus KO MEF. Similar to primary MEF derived from Acss2 knockout (KO) mice, MEF generated from Acss2 PM or Acss2 ED mice demonstrated markedly reduced endogenous Acss2 protein levels relative to MEF derived from Acss2 wild-type (WT) mice (Fig. 10*B*). The reduced expression of endogenous Acss2 protein in KO, PM, and ED mutant MEF resulted in reduced acetate-derived lipid synthesis (Fig. 10*C*).

We next addressed the potential reasons for reduced levels of endogenous Acss2 PM and ED protein in MEF. No significant differences were observed in the insoluble relative to soluble fraction of Acss2 PM and ED protein in comparison to Acss2 WT protein (Fig. 10*D*). Inhibition of protein translation with cycloheximide revealed that the half-life of Acss2 PM or ED proteins (estimated at between 4 and 8 h) is at least three times shorter than that of Acss2 WT protein (greater than 24 h), which indicates that Acss2 PM and ED proteins have reduced stability (Fig. 10*E*). Finally, degradation of Acss2 PM or ED proteins was not affected by inhibition of ubiquitin-mediated proteasomal degradation with MG132 (Fig. 10*F*) or by inhibition of autophagy with chloroquine (Fig. 10*G*).

## Discussion

We previously identified two potent PDEs in the Acss2 hinge region, the DHR, and the ABC domain (8). The ABC domain is an extremely potent PDE and is the most evolutionarily conserved element of the ACS superfamily. Herein, we performed an extensive exploration of the hinge region to identify additional PDE. We identified up to four additional PDEs: the A8 region that exhibits both PDE and masking traits; a potent PDE, the β-E2 region; and two PDE downstream of the ABC domain, which when present together constitute a conditional masked PDE (CMP). We also determined that the ABC domain contains two nonoverlapping PDE. In conjunction with the DHR and ABC domain, potential PDE comprise nearly three-quarters of the hinge region.

One emerging characteristic for the PDE elements in Acss2 is their short length, but otherwise they lack any clearly defining features. This is a theme that also characterizes degrons recognized by the anaphase-promoting complex, which polyubiquitinates specific cell cycle proteins at precise times determined in part by unique aspects of a given degron (21). The β-E2 region is at most 13 amino acids in length and is predicted to comprise part of a beta sheet. The DHR is 22 amino acids in length and includes part of an alpha helical region and a beta sheet, but likely is largely disordered in Acss2 homologs (10, 11, 22). Amino terminal truncations reduce DHR PDE function, but the effect of carboxy terminal truncations is unknown. The ABC domain defined by evolutionarily conserved residues is 18 amino acids in length (8). We demonstrate that the ABC domain can be divided into two segments, each of which functions as a potent PDE. Hence, motifs as short as nine amino acids exhibit potent PDE function. Some of these motifs are located in disordered segments of the Acss2 protein and may function as short linear motifs, particularly if these regions undergo modifications or serve as ligands for other cellular factors (23).

Other elements in the Acss2 hinge region are noteworthy. A8 exhibits both PDE and masking activities, the latter likely related to the presence of an acidic contact point that interacts with two basic contact points in the ABC domain. The A8 element is located at the most amino terminal portion of the Acss2 hinge region. Crystal structure of a prokaryotic Acss2 homologue indicates that it undergoes a conformational change during enzymatic generation of acetyl CoA involving rotation around the aspartic acid residue that marks the start of the hinge region. If similar conformational changes occur with mammalian Acss2, it is possible that its ability to function as either a PDE or masking element may be related to the spatial orientation of A8 in one of these conformations.

The PDEs located downstream of the ABC domain are also noteworthy. The amino terminal segment is at most 15 amino acids in length and is predicted to be an unstructured feature in Acss2 based upon corresponding segments in crystal structures of ACS proteins. The carboxy terminal segment is likewise 15 amino acids in length and is predicted to be an alpha-helical feature. These two segments function as a conditional PDE when present in isolation or in the absence of each other. However, when present together, these two segments effectively mask each other. Given the likely relationship of these two segments in the native Acss2 protein, we refer to this region as a conditionally masked PDE (CMP). Although we have not explored its molecular determinants, the CMP may be a conditional C-degron (24) given its location at the carboxy terminus, whereas the β-E2, DHR, and ABC domains may represent internal degrons. Whether the elements that constitute the carboxy terminal CMP are subject to dynamic changes through binding of other cellular factors or by PTMs that mask or unmask the PDE remains to be determined (25).

We have identified PDE by their ability to confer instability to a heterologous long-lived protein, moxGFP, the first defining feature of degrons. In some cases, we confirmed the ability of elements to function as PDE by deleting them from the parental Acss2 protein. Because we defined these elements functionally by fusing them to heterologous reporters or by deleting them from the parental Acss2 protein in order to interrogate their effect on protein stability, it is possible that some of these findings are artifactual. However, we argue that the PDEs identified in this study are *bona fide* elements for several reasons. First, the existence of multiple PDEs is supported by the observation that the ED substitution mutation in full-length Acss2 results in reduced protein stability, whereas this same mutation in the isolated ABC domain impairs its ability to function as a PDE. Second, the PM truncating mutation cleaves off the carboxy terminal CMP, which may mask the ABC domain and other PDE and exposes the amino portion of the ABC domain, which we contend is a potent internal PDE. Finally, the destabilizing effect of amino acid substitutions for putative electrostatic contact points in the Acss2 hinge domain as well as of hydrophobic residues in the ABC domain is profound. We propose that these residues provide critical structural contributions that maintain the hinge region in a tertiary conformation to mask its PDE, which is further supported by the highly evolutionarily conserved nature of these residues.

We found mutations that eliminated protein-destabilizing activity of the isolated PDE, the second defining feature of degrons. However, the molecular determinants of each PDE in the Acss2 hinge region appear to be distinct. For β-E2, it is likely that a major determinant is a structural aspect of the element. This conclusion is supported by our observation that the corresponding β-E2 regions from *S. cerevisiae*, *H. salinarium*, and *M. bovis* are potent PDEs despite the fact that their primary sequence deviates substantially from the mouse Acss2 β-E2 element, particularly the *M. bovis* β-E2 element. The scrambled mutant for β-E2 is stable, which suggests that there may be positional requirements for some amino acids. However, the limitation for scrambled mutants is that they may lose any conserved structural feature, whereas this is less likely for substitutions that use similar motifs from Acss2 homologues in other species.

The results from mutagenesis of the DHR revealed several interesting findings. Similar to the β-E2 mutagenesis experiment, substitutions with homologous elements from several species, *S. cerevisiae* and *H. salinarium*, reduced the ability of this region to function as a PDE, whereas the DHR from *D. radiodurans* and *M. bovis* did not impair PDE function. The DHR scrambled mutant also exhibited enhanced stability, although to a lesser extent than DHR from *S. cerevisiae* and *H. salinarium*. The enhanced stability of the DHR from *S. cerevisiae* was surprising given this region in mouse Acss2 was originally identified by virtue of its homology to this element. However, Acss2 homologues from *S. cerevisiae* and other lower eukaryotes contain a stretch of amino acids upstream of the DHR that is not present in other Acss2 homologues, which may alter the boundaries of the DHR in these organisms. Whether the lack of an effect of the *S. cerevisiae* DHR on protein stability in this study is a result of species-specific differences in recognition of this element, incomplete reconstruction of the *S. cerevisiae* DHR element, or altered amino acids upstream of the DHR defined by junctions with exogenous reporters remains to be addressed (13).

The ABC domain remains a complicated element in Acss2 and was the PDE most resistant to mutational inactivation. Because a loosely associated characteristic of some degrons is enrichment in basic residues, we performed an extensive mutagenesis targeting basic residues in the ABC domain. Pairwise as well as complete alanine substitutions for basic residues in the ABC domain had no effect on isolated PDE function. Scrambled mutations also did not affect isolated PDE function. The only mutations we found that inactivate isolated ABC domain PDE function were acidic substitutions for one or two basic residues in the ABC domain, which include the Acss2 ED substitution mutation (8). The ED substitution mutation impairs the ability of Acss2 to translocate into the nucleus when expressed in transformed cells (7), but dramatically reduces endogenous Acss2 protein levels in mice (8).

Residues in the ABC domain undergo PTMs. Acetylation of a lysine residue affects enzymatic activity in ACS (12, 26) as well as in Acss2 (17, 18), and phosphorylation of a serine residue may regulate Acss2 nuclear translocation (15). None of the PTM mimetics altered the PDE activity of the isolated ABC domain, although they may affect full-length Acss2 protein levels. The phosphorylation mimetic, S659D, and acetylation mimetic, K661Q, increase, whereas the dephosphorylation mimetic, S659A, and deacetylation mimetic, K661R, have no effect on ectopically expressed full-length Acss2 protein levels in KO MEF. Another deacetylation mimetic, K661A, also has no effect on Acss2 protein levels in these assays. Whether these patterns are maintained if the mimetics are expressed in the context of endogenous Acss2 is unknown.

We propose that conservation of the basic and acidic contact residues in the ABC domain reflects their importance in maintaining tertiary structure of the hinge region. Two basic contacts in the ABC domain and their acidic contacts in A8 and α − 17 elements are conserved across prokaryotes and eukaryotes. Pairwise alanine substitutions for these basic residues result in unstable full-length Acss2 protein. The third basic contact in the ABC domain and its acidic contact in the CMP is less conserved, although these residues are conserved in higher organisms. Single alanine substitutions for these latter residues have no or minimal effect on full-length Acss2 protein stability. Prokaryotic Acss2 homologues have shorter carboxy terminal sequences, which may render this contact pair less essential.

The importance of electrostatic contacts in maintaining masking of PDEs in Acss2 is demonstrated by the effect of the ED mutation on stability of the moxGFP:isolated ABC domain fusion versus its effect on stability of the full-length Acss2 ED protein. Although moxGFP fused to the isolated ED ABC domain is stable, full-length Acss2 ED protein is unstable. We speculate that the ED mutation disrupts at least two of three electrostatic interactions linking the ABC domain with masking elements in the Acss2 hinge region, thereby exposing the remaining PDE. However, hydrophobic interactions are also likely important in masking of the ABC domain and other PDE. Leucine, isoleucine, proline and glycine residues in the ABC domain are critical for stability of full-length Acss2 protein and are highly conserved in the Acss2 superfamily as are the serine and lysine residues subject to PTM. Thus, the evolutionary constraints to maintain these electrostatic contact, hydrophobic, and PTM residues in the ABC domain offer a rationale for why this domain is the most highly conserved element of the Acss2 superfamily.

One potential role for the existence of multiple PDE in Acss2 is in protein quality control (27). The Acss2 hinge region contains domains essential for its enzymatic function (10, 11, 12). Although separated in primary sequence, the PDEs in the Acss2 hinge region reside in close proximity to each other in the tertiary state and contain evolutionarily conserved residues. Several PDEs are constituted in whole or in part by unstructured regions. These may assume more specific conformations depending upon the enzymatic status, cellular metabolic state (28, 29), or PTM status (30, 31). We note that in this study, posttranslational mimetics of the ABC domain did not eliminate its PDE function.

Acss2 may contain multiple PDEs in order to ensure that a malfunctioning protein does not act as a sink for limiting substrates or cofactors. A recent study described a transition-state mimetic that binds Acss2 in the amino globular region and impedes Acss2 function in breast cancer (32). However, its effect on Acss2 protein stability was not reported. It is possible that Acss2 protein stability is affected by natural compounds or metabolites binding in enzymatic pockets that do not progress in the enzymatic conversion and instead lock Acss2 in a state that exposes one or more PDEs, potentially in a synergistic manner (33). Thus, the possibility that PDEs act as dynamic sensors of the cellular biochemical state remains an intriguing possibility.

The third step in formally designating a PDE as a degron is association with a proteolytic mechanism (34), typically ubiquitin-dependent proteasomal degradation with degrons serving as docking sites for E3 ligases. Ectopic Acss2 PM and ED proteins expressed in MEFs exhibit lower steady-state levels than ectopic Acss2 WT protein. Endogenous Acss2 PM and ED proteins produced in MEF derived from Acss2 PM and ED mice are also unstable, but this is not due to insoluble aggregates, one mechanism for protein removal (35, 36). Endogenous Acss2 PM and ED proteins have a reduced half-life, but are not degraded by the proteasome or by autophagy. We could not discern any obvious PEST sequences (37) or motifs for lysosomal targeting or chaperone-mediated autophagy (38, 39) in the PDE. Ubiquitination-independent (40, 41, 42, 43, 44) as well as proteasome-independent (45) pathways for protein degradation have been described (46). Interestingly, ectopic Acss2 PM and ED protein levels are higher when expressed in Acss2 WT versus KO MEF. They are also higher when expressed in transformed versus primary cells. Whether these higher levels are due to saturation of cellular degradation pathways that target Acss2, stabilizing interactions of mutant Acss2 proteins with Acss2 WT protein, or a consequence of the transformed state remains to be determined.

Rotation of the carboxy terminus of ACS following the first step of acetyl CoA generation and prior to the final step of the acetyl CoA generation (10, 11) may expose hidden surfaces in the hinge region. If this occurs for eukaryotic Acss2, it potentially unmasks an NLS as the ABC domain fits a consensus sequence for an NLS. Acss2 can transit or be enriched in the nucleus following oxygen or glucose deprivation (2, 7, 15), and other events may also regulate Acss2 subcellular localization. However, the behavior of Acss2 protein in transformed cell lines may differ from its behavior in primary, nonmalignant cells. Introduction of a candidate cytosol-restricting mutation, the ED mutation, into the germline of mice resulted in severely reduced Acss2 ED protein levels and subsequently led to the identification of the ABC as a potent PDE (8).

Recent studies in yeast have identified degrons that direct cytosolic and nuclear proteins for degradation (13, 47). Interestingly, the presence of an NLS may have a significant effect on degron potency (13). We have not confirmed that the ABC domain regulates Acss2 subcellular localization. This is a challenge for several reasons including that many substitutions in the ABC domain destabilize the full-length protein and fusion proteins containing the isolated ABC domain are efficiently degraded. Future studies will be required to identify the mechanism by which unstable Acss2 proteins are eliminated from the cell as well as to assess how Acss2 subcellular localization is regulated.

We conclude by emphasizing functional consequences for premature truncations of Acss2. The Acss2 PM truncating mutation has a profound effect on Acss2 protein stability in mice, similar to the Acss2 ED substitution mutant (8). It exposes the amino terminal ABC domain, which can function as a potent PDE, and eliminates all basic contact points with masking elements. Since Acss2 is a key component of the HIF-2 signal transduction pathway (2, 7, 9), we investigated whether mice harboring the Acss2 PM mutation have altered HIF-2 signaling. Indeed, mice with homozygous PM alleles have virtually absent Acss2 protein levels and blunted HIF-2 signaling activated by acute anemia, similar to mice with the Acss2 ED substitution mutation (8). Mouse embryonic fibroblasts isolated from Acss2 PM and ED mice have severely reduced levels of acetate-derived lipids compared with mouse embryonic fibroblasts isolated from Acss2 WT mice, which likely will manifest as reduced acetate-dependent lipid synthesis in Acss2 PM and ED mice. Thus, while the functional role of the ABC domain and other PDE in the native Acss2 protein remains speculative, it is evident that truncating and substitution variants in the Acss2 hinge region have severe consequences for Acss2-related biology, which may be of relevance to human disease (48).

## Experimental procedures

### Stable cell culture studies

#### Cell culture

Transformed cell lines used in this study included HEK293T (lentivirus generation) and HeLa (stable cell lines). All cells were maintained in a cell culture incubator at 37 °C, 5% CO_2_, and 95% air and passaged as previously described in detail (8). Standard cell culture media consisted of DMEM (Corning, Cat. No. 10-013-CV), 10% fetal bovine serum (Atlanta Biologicals, Cat. No. S11150), and 1× Penicillin/Streptomycin (Corning, Cat. No. 30-002-CI). To passage, cells were trypsinized with 0.25% trypsin-EDTA (Corning, Cat. No. 25-053-CI) and split at a ratio of 1:6 (HeLa) or 1:10 (HEK293T).

#### Expression constructs

Lentiviruses expression constructs were made as previously described in detail (8). Briefly, moxGFP (Addgene, Cat. No. 68070) was cloned into a customized pLenti6/V5-GW/lacZ-based transfer cassette (Invitrogen). Indicated regions of Acss2 were joined in-frame with the moxGFP carboxy terminus by restriction sites or by zipper PCR. Full-length mouse Acss2 with an amino terminal V5 epitope tag was cloned into either pIRES-hrGFP-2A (Agilent Technologies, Cat. No. 240032) or a pLenti6/V5-GW/lacZ-based transfer cassette (Invitrogen).

#### Lentivirus production and stable cell line generation

Lentiviruses and stable cell lines were made as previously described (8). Briefly, pLenti6-based (Invitrogen) expression constructs were cotransfected into HEK293T cells with packaging plasmids psPAX2 (Addgene, Cat. No. 12260) and pMD2.G (Addgene, Cat. No. 12259) using polyethylenimine (PEI; Polysciences, Cat. No. 23966-1) at a ratio of 3.5 μg PEI/1 μg DNA. Media was changed 12 h after transfection, followed by two collections of virus-containing supernatant 24 and 48 h after the first media change. Supernatant was subjected to ultracentrifugation at 100,000*g* (24,000 rpm) in an SW28 rotor for 2 h at 4 °C and the virus pellet resuspended overnight in 1 ml PBS. To generate stably integrated cell lines, HeLa was incubated with lentivirus for 24 h, cells were incubated with media containing 5 μg/ml Blasticidin S (Invivogen, Cat. No. ant-bl-1) for 4 days, followed by 2 weeks of selection at a concentration of 2 μg/ml Blasticidin S. Cells used in experiments were plated in media without Blasticidin S.

#### Protein extract preparation and immunoblotting

Whole cell extracts and immunoblotting were performed as described earlier (8). Soluble proteins were prepared by centrifugation of cell lysates for 15 min in a microfuge at 4 °C, 18,000*g*. To assay insoluble proteins, remaining cell pellets were resuspended in solubilization buffer (20 mM phosphate buffer pH 8.0, 300 mM NaCl, 2% SDS, 2 mM DTT, 1% Triton X-100, 8 M urea, and 1× protease inhibitor cocktail) and incubated at room temperature for 5 min, followed by centrifugation at 18,000*g* for 15 min at 4 °C in a microfuge. The supernatant was then heated with in sample buffer (Bio-Rad, Cat. No. 1610737) for 5 min at 37 °C. Antibodies recognizing the following epitopes were used for immunoblotting at the indicated dilutions: Acss2, 1:1000 (Cell Signaling Technology, Cat. No. 3658); GFP, 1:1000 (Invitrogen, Cat. No. A11121); α-tubulin, 1:5000 (Sigma, Cat. No. T9026); V5, 1:1000 (Invitrogen, Cat. No. R96025); LC3B, 1:1000 (Cell Signaling Technology, Cat. No. 2775); ubiquitin, 1:1000 (Cell Signaling Technology, Cat. No. 3936); HRP-linked anti-mouse IgG (Cell Signaling Technology, Cat. No. 7076); HRP-linked anti-rabbit IgG (Cell Signaling Technology, Cat. No. 7074).

### Bioinformatics

#### Homology comparisons

Acss2 protein sequences from animals were identified manually from the NCBI or UniProt database and the protein identifiers for each organism are provided for review (Table S1). Protein sequences were initially examined for alignment to a consensus sequence. When significant deviation occurred, raw genomic sequence files for predicted proteins were examined for potential frame shifts and corrections were introduced to restore alignment to the consensus sequence. Final edited protein sequences were aligned and compared using Clustal Omega (19, 20). In the alignments, an asterisk (∗) indicates complete conservation across all organisms; a colon (:) indicates conservation between groups of amino acids with strongly similar properties, which are roughly equivalent to scores of greater than 0.5 in the Gonnet PAM 250 matrix; and a period (.) indicates conservation between groups of amino acids with weakly similar properties, which are roughly equivalent to scores of equal to or less than 0.5 in the Gonnet PAM 250 matrix.

#### Molecular modeling

The mouse Acss2 WT protein was modeled using a web-based program, SWISS-MODEL (49). Default parameters were used in modeling predictions with the SWISS-MODEL server homology modeling pipeline in conjunction with the repository comprising the SWISS-MODEL template library. The template with highest predicted correlation based upon the Global Model Quality Estimation (GMQE) and QMEAN Z-score was used for comparison. The SWISS-MODEL pipeline identified prokaryotic Acss2 homologs as template models for mouse Acss2 protein with the crystal structure of acetyl-coenzyme A synthetase containing an R194A mutation as the highest match (SMTL ID: 2p2m.1) (10). The GMQE score for mouse Acss2 protein is 0.76 with a QMEAN Z-score of –1.20.

### Whole animal studies

#### Mutant mouse generation

The mouse Acss2 gene was targeted for CRISPR/Cas9 by the University of Texas Southwestern Transgenic Core to generate Acss2 ED mice as described earlier (8). When characterizing Acss2 ED founder mice, we determined that one line contained a point mutation (single nucleotide deletion) in the ABC domain, which introduces a stop codon following addition of one nonnative amino acid residue. We designated this mouse line Acss2 PM for point mutation. After back-crossing Acss2 PM founder mice for several generations with C57BL/6J mice, heterozygous mating pairs generated homozygous Acss2 PM mice and WT litter-mates. Mice were maintained in standard bedding with microisolator caging under germ-free conditions at animal facilities located at the University of Texas Southwestern, fed and watered *ad lib* throughout the study, and monitored for general health with sentinel mice. All mice used in experiments were generated from breeding colonies in animal facilities located at the University of Texas Southwestern. All animal experiments were approved by the University of Texas Southwestern Institutional Animal Care and Use Committee (APN 2016-101616).

#### Immunohistochemistry and immunoblot studies

Kidney and liver samples from Acss2 WT and PM adult mice were formalin-fixed, paraffin-embedded, and used for Acss2 immunohistochemical analysis as previously described (9). Protein samples were prepared from the liver, kidney, brain, heart, lung, and spleen samples (10 μg) as well as from MEF using CytoBuster protein extraction reagent (Novagen, Cat. No. 71009) with 1× protease inhibitor cocktail (Sigma, Cat. No. P8340) as previously described (8). Extracts were electrophoresed and subjected to immunoblot analysis as previously described (8) with primary antibodies for the following antigens: human Acss2, 1:1000 (Cell Signaling Technology, Cat. No. 3658); α-tubulin, 1:3000–1:5000 dilution (Sigma, Cat. No. T9026).

#### Anemia studies

Acute, hemolytic anemia was induced in mice by phenylhydrazine (PHZ; Sigma, Cat. No. 114715) injection as previously described (8). Four days after the first PHZ injection, spun hematocrits were determined in duplicate with blood obtained retro-orbitally. Mice with hematocrits within the target range of 19–27% were used for experimental analysis. Hematocrits for individual mice are available for review (Table S2).

#### Molecular studies

Mouse kidney and liver total RNAs were prepared from individual organs using the FastRNA Pro Green kit (MP Biomedicals, Cat. No. 116045050) on the FastPrep-24 bead grinder and lysis system (MP Biomedicals, Cat. No. 116004500). Mouse kidney and liver cDNAs were prepared from 1 μg RNA with the iScript cDNA Synthesis kit (Bio-Rad, Cat. No. 1708891) for mEpo measurements. Mouse erythropoietin (mEpo), Acss2 (mAcss2), and cyclophilin B (mCyclB) mRNA levels were measured in duplicate or triplicate by real-time PCR (rtPCR) semiquantitative analyses as previously described (8) from 10 ng of total RNA per reaction with the following modifications. Real-time PCR was performed on an ABI Prism 7900HT thermocycler in a 384-well format using Power SYBR Green Master Mix (ThermoFisher, Cat. No. 4368577). Primers were obtained from a commercial vendor (IDT). Primers and cycling parameters were adjusted to optimize Epo and Acss2 mRNA measurements from each organ by examination of melting curves. For kidney mEpo mRNA measurements, amplification of cDNA was performed with 45 cycles of a two-step PCR with denaturation at 95 °C for 15 s and annealing/extension at 60 °C for 45 s using the following primers: mEpo.gsrt.139.for6: 5′-GAGGCAGAAAATGTCACGATG-3′, mEpo.gsrt.250.rev6: 5′-CTTCCACCTCCATTCTTTTCC-3′. For liver mEpo mRNA measurements, amplification of cDNA was performed with 45 cycles of a two-step PCR with denaturation at 95 °C for 15 s and annealing/extension at 63 °C for 30 s using the following primers: mEpo.jgrt.for.11: 5′-GGAGGCAGAAAATGTCACGATG-3′, mEpo.jgrt.rev.11: 5′-CTGTTCTTCCACCTCCATTC-3′. For mAcss2 mRNA measurements from the kidney and liver, amplification of cDNA was performed with 40 cycles of a two-step pcr with denaturation at 95 °C for 15 s and annealing/extension at 60 °C for 60 s using the following primers: mAcss2.jgrt.for1: 5′- AAACACGCTCAGGGAAAATCA-3′, mAcss2.jgrt.rev1: 5′- ACCGTAGATGTATCCCCCAGG-3′. The results of duplicate (cyclophilin B for some tissues) or triplicate experiments were expressed as two exponential (-(mEpo or mAcss2 number of cycles-cyclophilin number of cycles)). The normalizer in each set was WT control samples except for liver mEpo measurements. In this case, nearly all WT samples except for one were undetectable, whereas there were detectable, but low, measurements of mEpo in several liver PM samples, which was therefore used as a normalizer. For individual mouse samples where the majority of samples were undetectable, a relative value of 0.1 was assigned as a floor value. Raw PCR data are available for review (Table S2).

### Primary mouse embryonic fibroblast studies

#### Mouse embryonic fibroblast preparation and nucleofection

MEFs were derived from Acss2 ED, PM, and KO mouse matings as described earlier (8). MEFs from homozygous Acss2 WT, ED, PM, or KO embryos were expanded twice and frozen in aliquots. Nucleofection of MEFs was performed using a Lonza 4D-Nucleofector (Lonza, Cat. No. AAF-1002B). Individual vials of frozen, low-passage Acss2 WT or KO MEFs were plated for experiments 2 days before nucleofection. For each construct, 750,000 cells were nucleofected with 2 μg endotoxin-free expression plasmid using the Amaxa P4 Primary Cell 4D-Nucleofector X Kit (Lonza, Cat. No. V4XP-4024) and manufacturer program CZ-167. After nucleofection, the cells were transferred to a preincubated 6-well plate containing 2 ml of media per well and returned to the incubator. Media was changed after 6 h. Cells were harvested 2 days postnucleofection.

#### Endogenous Acss2 protein studies with mouse embryonic fibroblasts

Acss2 WT, PM homozygous, and ED homozygous MEFs were plated onto 6-well plates 2 days before the start of treatment. Media was changed to standard cell culture media plus vehicle 24 h before harvest. At 24, 12, 8, 4, and 2 h before harvest, media on one well of each genotype was replaced with standard cell culture media plus inhibitor. At harvest, cells including an untreated, 0-h control were harvested and immunoblotted as described earlier with the following modification: protein extract buffer was supplemented with the inhibitor used for each experiment at the same concentration used for treatment. The following inhibitors were used at the indicated final concentrations: 50 μg/ml cycloheximide (Cayman Chemical, Cat. No. 14126); 1 μM MG132 (UBP Bio, Cat. No. F1100); 50 μM chloroquine (Sigma, Cat. No. C6628). Stock solutions of cycloheximide and MG132 were prepared in DMSO under sterile conditions. Chloroquine was prepared in molecular grade water and filter-sterilized.

#### Acetate-dependent lipid synthesis studies with mouse embryonic fibroblasts

Acss2 WT, PM homozygous, and ED homozygous MEFs were plated onto 6-well plates. Media was changed 6 h before treatment. For 24-h acetate labeling, 5.0 μl (0.5 μCi) of [1,2-^14^C] acetate (PerkinElmer, Cat No. NEC553250UC) was added to wells in triplicate 24 h before harvest. The following day, one untreated well of each genotype was trypsinized, pelleted, and frozen for protein quantification and western blotting. For the 0-h labeling control, 5.0 μl labeled acetate was added to three wells of WT control cells and swirled to mix, followed by immediate harvest. Each well was rinsed with PBS. Cells were then scraped into 2 ml of PBS and transferred to a borosilicate glass tube. The cells were pelleted at 750*g* for 5 min at room temperature. The PBS was removed and cell pellet resuspended in 400 μl 0.5% Triton X-100 in water. The following were added to each lysate, followed by 15 s vortexing after each addition: 2 ml methanol, 2 ml chloroform, 1 ml water. The phases were separated by centrifugation at 900*g* for 10 min. The lower, organic layer was transferred to a new tube with a Pasteur pipette. The aqueous mixture was re-extracted with 2 ml chloroform, adding the organic layer to the first extraction, and dried under a nitrogen stream. Dried lipid extract was resuspended in 200 μl chloroform and transferred to a scintillation vial containing 3 ml Ecolume (MP Biomedicals, Cat No. 0188247001). Scintillation counts per minute were read on a Beckman LS6500. Counts were normalized to protein content determined with aliquots obtained in parallel.

## Data availability

Data described in this study are contained within the manuscript or supporting information.

## Supporting information

This article contains supporting information.

## Conflict of interest

The authors declare that they have no conflicts of interest with the contents of this article.

## References

[1] Pietrocola F., Galluzzi L., Bravo-San Pedro J.M., Madeo F., Kroemer G. (2015). Acetyl coenzyme A: A central metabolite and second messenger. Cell Metab..

[2] Chen R., Xu M., Nagati J.S., Hogg R.T., Das A., Gerard R.D., Garcia J.A. (2015). The acetate/ACSS2 switch regulates HIF-2 stress signaling in the tumor cell microenvironment. PLoS One.

[3] Chen R., Xu M., Hogg R.T., Li J., Little B., Gerard R.D., Garcia J.A. (2012). The acetylase/deacetylase couple CREB-binding protein/Sirtuin 1 controls hypoxia-inducible factor 2 signaling. J. Biol. Chem..

[4] Dioum E.M., Chen R., Alexander M.S., Zhang Q., Hogg R.T., Gerard R.D., Garcia J.A. (2009). Regulation of hypoxia-inducible factor 2alpha signaling by the stress-responsive deacetylase sirtuin 1. Science.

[5] Hu A., Yang L.Y., Liang J., Lu D., Zhang J.L., Cao F.F., Fu J.Y., Dai W.J., Zhang J.F. (2020). SIRT2 modulates VEGFD-associated lymphangiogenesis by deacetylating EPAS1 in human head and neck cancer. Mol. Carcinog.

[6] Luong A., Hannah V.C., Brown M.S., Goldstein J.L. (2000). Molecular characterization of human acetyl-CoA synthetase, an enzyme regulated by sterol regulatory element-binding proteins. J. Biol. Chem..

[7] Chen R., Xu M., Nagati J., Garcia J.A. (2017). Coordinate regulation of stress signaling and epigenetic events by Acss2 and HIF-2 in cancer cells. PLoS One.

[8] Nagati J.S., Xu M., Garcia T., Comerford S.A., Hammer R.E., Garcia J.A. (2019). A substitution mutation in a conserved domain of mammalian acetate-dependent acetyl CoA synthetase 2 results in destabilized protein and impaired HIF-2 signaling. PLoS One.

[9] Xu M., Nagati J.S., Xie J., Li J., Walters H., Moon Y.A., Gerard R.D., Huang C.L., Comerford S.A., Hammer R.E., Horton J.D., Chen R., Garcia J.A. (2014). An acetate switch regulates stress erythropoiesis. Nat. Med..

[10] Reger A.S., Carney J.M., Gulick A.M. (2007). Biochemical and crystallographic analysis of substrate binding and conformational changes in acetyl-CoA synthetase. Biochemistry.

[11] Gulick A.M., Starai V.J., Horswill A.R., Homick K.M., Escalante-Semerena J.C. (2003). The 1.75 A crystal structure of acetyl-CoA synthetase bound to adenosine-5'-propylphosphate and coenzyme A. Biochemistry.

[12] Starai V.J., Gardner J.G., Escalante-Semerena J.C. (2005). Residue Leu-641 of Acetyl-CoA synthetase is critical for the acetylation of residue Lys-609 by the Protein acetyltransferase enzyme of Salmonella enterica. J. Biol. Chem..

[13] Geffen Y., Appleboim A., Gardner R.G., Friedman N., Sadeh R., Ravid T. (2016). Mapping the landscape of a eukaryotic degronome. Mol. Cell.

[14] Bulusu V., Tumanov S., Michalopoulou E., van den Broek N.J., MacKay G., Nixon C., Dhayade S., Schug Z.T., Vande Voorde J., Blyth K., Gottlieb E., Vazquez A., Kamphorst J.J. (2017). Acetate recapturing by nuclear acetyl-CoA synthetase 2 prevents loss of histone acetylation during oxygen and serum limitation. Cell Rep..

[15] Li X., Yu W., Qian X., Xia Y., Zheng Y., Lee J.H., Li W., Lyu J., Rao G., Zhang X., Qian C.N., Rozen S.G., Jiang T., Lu Z. (2017). Nucleus-translocated ACSS2 promotes gene transcription for lysosomal biogenesis and autophagy. Mol. Cell.

[16] Costantini L.M., Baloban M., Markwardt M.L., Rizzo M., Guo F., Verkhusha V.V., Snapp E.L. (2015). A palette of fluorescent proteins optimized for diverse cellular environments. Nat. Commun..

[17] Hallows W.C., Lee S., Denu J.M. (2006). Sirtuins deacetylate and activate mammalian acetyl-CoA synthetases. Proc. Natl. Acad. Sci. U. S. A..

[18] Sahar S., Masubuchi S., Eckel-Mahan K., Vollmer S., Galla L., Ceglia N., Masri S., Barth T.K., Grimaldi B., Oluyemi O., Astarita G., Hallows W.C., Piomelli D., Imhof A., Baldi P. (2014). Circadian control of fatty acid elongation by SIRT1 protein-mediated deacetylation of acetyl-coenzyme A synthetase 1. J. Biol. Chem..

[19] Sievers F., Higgins D.G. (2018). Clustal Omega for making accurate alignments of many protein sequences. Protein Sci..

[20] Sievers F., Wilm A., Dineen D., Gibson T.J., Karplus K., Li W., Lopez R., McWilliam H., Remmert M., Soding J., Thompson J.D., Higgins D.G. (2011). Fast, scalable generation of high-quality protein multiple sequence alignments using Clustal Omega. Mol. Syst. Biol..

[21] Davey N.E., Morgan D.O. (2016). Building a regulatory network with short linear sequence motifs: Lessons from the degrons of the anaphase-promoting complex. Mol. Cell.

[22] Jogl G., Tong L. (2004). Crystal structure of yeast acetyl-coenzyme A synthetase in complex with AMP. Biochemistry.

[23] van der Lee R., Buljan M., Lang B., Weatheritt R.J., Daughdrill G.W., Dunker A.K., Fuxreiter M., Gough J., Gsponer J., Jones D.T., Kim P.M., Kriwacki R.W., Oldfield C.J., Pappu R.V., Tompa P. (2014). Classification of intrinsically disordered regions and proteins. Chem. Rev..

[24] Varshavsky A. (2019). N-degron and C-degron pathways of protein degradation. Proc. Natl. Acad. Sci. U. S. A..

[25] Sharma S., Toledo O., Hedden M., Lyon K.F., Brooks S.B., David R.P., Limtong J., Newsome J.M., Novakovic N., Rajasekaran S., Thapar V., Williams S.R., Schiller M.R. (2016). The functional human C-terminome. PLoS One.

[26] Starai V.J., Celic I., Cole R.N., Boeke J.D., Escalante-Semerena J.C. (2002). Sir2-dependent activation of acetyl-CoA synthetase by deacetylation of active lysine. Science.

[27] Fredrickson E.K., Gardner R.G. (2012). Selective destruction of abnormal proteins by ubiquitin-mediated protein quality control degradation. Semin. Cell Dev. Biol..

[28] Dyson H.J., Wright P.E. (2005). Intrinsically unstructured proteins and their functions. Nat. Rev. Mol. Cell Biol..

[29] Davey N.E. (2019). The functional importance of structure in unstructured protein regions. Curr. Opin. Struct. Biol..

[30] Ravid T., Hochstrasser M. (2008). Diversity of degradation signals in the ubiquitin-proteasome system. Nat. Rev. Mol. Cell Biol..

[31] Bah A., Forman-Kay J.D. (2016). Modulation of intrinsically disordered protein function by post-translational modifications. J. Biol. Chem..

[32] Miller K.D., Pniewski K., Perry C.E., Papp S.B., Shaffer J.D., Velasco-Silva J.N., Casciano J.C., Aramburu T.M., Srikanth Y.V.V., Cassel J., Skordalakes E., Kossenkov A.V., Salvino J.M., Schug Z.T. (2021). Targeting ACSS2 with a transition state mimetic inhibits triple-negative breast cancer growth. Cancer Res..

[33] Guharoy M., Bhowmick P., Sallam M., Tompa P. (2016). Tripartite degrons confer diversity and specificity on regulated protein degradation in the ubiquitin-proteasome system. Nat. Commun..

[34] Varshavsky A. (1991). Naming a targeting signal. Cell.

[35] Sontag E.M., Vonk W.I.M., Frydman J. (2014). Sorting out the trash: The spatial nature of eukaryotic protein quality control. Curr. Opin. Cell Biol..

[36] Hipp M.S., Park S.H., Hartl F.U. (2014). Proteostasis impairment in protein-misfolding and -aggregation diseases. Trends Cell Biol..

[37] Rechsteiner M., Rogers S.W. (1996). PEST sequences and regulation by proteolysis. Trends Biochem. Sci..

[38] Tekirdag K., Cuervo A.M. (2018). Chaperone-mediated autophagy and endosomal microautophagy: Joint by a chaperone. J. Biol. Chem..

[39] Dice J.F. (1990). Peptide sequences that target cytosolic proteins for lysosomal proteolysis. Trends Biochem. Sci..

[40] Takeuchi J., Chen H., Hoyt M.A., Coffino P. (2008). Structural elements of the ubiquitin-independent proteasome degron of ornithine decarboxylase. Biochem. J..

[41] Takasugi T., Minegishi S., Asada A., Saito T., Kawahara H., Hisanaga S. (2016). Two degradation pathways of the p35 Cdk5 (cyclin-dependent kinase) activation subunit, dependent and independent of ubiquitination. J. Biol. Chem..

[42] Fortmann K.T., Lewis R.D., Ngo K.A., Fagerlund R., Hoffmann A. (2015). A regulated, ubiquitin-independent degron in IkappaBalpha. J. Mol. Biol..

[43] Shumway S.D., Miyamoto S. (2004). A mechanistic insight into a proteasome-independent constitutive inhibitor kappaBalpha (IkappaBalpha) degradation and nuclear factor kappaB (NF-kappaB) activation pathway in WEHI-231 B-cells. Biochem. J..

[44] Tao T., Shi H., Guan Y., Huang D., Chen Y., Lane D.P., Chen J., Peng J. (2013). Def defines a conserved nucleolar pathway that leads p53 to proteasome-independent degradation. Cell Res..

[45] Im S., Kim D.W. (2017). Nkx3.2 induces oxygen concentration-independent and lysosome-dependent degradation of HIF-1alpha to modulate hypoxic responses in chondrocytes. Cell Signal.

[46] Erales J., Coffino P. (2014). Ubiquitin-independent proteasomal degradation. Biochim. Biophys. Acta.

[47] Maurer M.J., Spear E.D., Yu A.T., Lee E.J., Shahzad S., Michaelis S. (2016). Degradation signals for ubiquitin-proteasome dependent cytosolic protein quality control (CytoQC) in yeast. G3 (Bethesda).

[48] Stein A., Fowler D.M., Hartmann-Petersen R., Lindorff-Larsen K. (2019). Biophysical and mechanistic models for disease-causing protein variants. Trends Biochem. Sci..

[49] Waterhouse A., Bertoni M., Bienert S., Studer G., Tauriello G., Gumienny R., Heer F.T., de Beer T.A.P., Rempfer C., Bordoli L., Lepore R., Schwede T. (2018). SWISS-MODEL: Homology modelling of protein structures and complexes. Nucleic Acids Res..

